# Impact of lamina-open side on unilateral open door laminoplasty in patients with degenerative cervical myelopathy

**DOI:** 10.1038/s41598-023-28490-7

**Published:** 2023-02-04

**Authors:** Kyung-Chung Kang, Sang-Kyu Im, Jung-Hee Lee, Ki Young Lee, Dong-Uk Seo, In-Uk Hwang

**Affiliations:** Department of Orthopaedic Surgery, College of Medicine, Kyung Hee University Hospital, Kyung HeeUniversity, Seoul, Republic of Korea

**Keywords:** Outcomes research, Musculoskeletal system

## Abstract

Surgeons should select one side for cervical unilateral open door laminoplasty (UODL). However, few reports suggest proper guidelines for deciding which side to open. The aim of this study is to evaluate the impact of opening side in UODL on dominant cord compressive or symptomatic side. 193 degenerative cervical myeloradiculopathy patients with followed-up more than 2 years were enrolled. In all cases, UODL was performed uniformly on the right side. Patients were sub-grouped based on preoperative dominant 3 characteristics: cord compression, myelopathy symptom and radiculopathy symptom (right, symmetric, left). Pre- and postoperative radiographic and clinical parameters and incidence of postoperative C5 palsy were analyzed and compared among the groups. According to dominant compressive side, there were no significant differences in postoperative radiographic and clinical parameters among three groups. According to dominant myelopathy or radiculopathy symptom side, there were no significant differences of all radiographic and clinical parameters postoperatively, except slightly lower neck VAS in groups of preoperative right dominant myelopathy or radiculopathy symptom side at postoperative 1 month. C5 palsies occurred in twelve patients (6.2%), but the incidences were not different among the groups. Therefore, when performing UODL, the choice of lamina opening side can be left to surgeon’s preference.

## Introduction

Cervical laminoplasty is one of world-widely used surgical procedures for multi-level degenerative cervical diseases, such as cervical spondylotic myelopathy (CSM) and ossification of posterior longitudinal ligament (OPLL)^1^. It has been considered an effective method to decompress multi-level cervical spinal cord compression^2,3^ and has various advantages, such as preservation of segmental stability and range of motion (ROM) or prevention of postoperative kyphosis and adjacent segment diseases after cervical fusion surgery^4,5^.

The unilateral open-door laminoplasty (UODL) was first introduced by Hirabayashi et al. in 1983^2^ and although there are controversial issues^3^, it is one of the most world-widely used laminoplasty technique due to its feasibility, short operation time and satisfactory long-term results^6,7^. This procedure enlarges the anterior–posterior diameter of the spinal canal by opening one side of the lamina to the opposite side. When performing the UODL, the surgeons should choose one direction of the lamina for the opening side. The surgeons usually select the side based on severe cord compression of the computed tomography (CT)/magnetic resonance imaging (MRI) or preoperative symptom. Some surgeons decide the opening side according to their own convenience. In the literatures, Hirabayashi et al. recommended opening the dominant symptomatic side in their original report^2^, but in recent two studies, Tang et al. and Shao et al. concluded that the contralateral side opening is preferable to the ipsilateral side opening in the UODL for the lateral type OPLL^8,9^.

As such, there is no clear standard for the selection of the lamina opening side in the UODL to date. The aim of this study is to evaluate the effect of the opening side of the lamina according to the dominant cord compressive side or symptomatic side on radiographic and clinical outcomes in patients undergoing the UODL and suggest a proper guideline for the selection of the lamina opening side.

## Methods

All procedures were indicated and performed in compliance with our department’s standards and the Declaration of Helsinki and every participant of this study provided written informed consent. This study was approved by the institutional review boards at Kyung Hee University Hospital (KHUH IRB: 2021–09-059).

### Patient selection and study design

This study was a retrospective review of consecutive patients with multi-level degenerative cervical myelopathy underwent the UODL at our institution from March 2015 to September 2019. Among 205 patients, the patients who had medical history of trauma, infection, infection or inflammatory disease and less than 2 years of postoperative follow-up were excluded. Finally, 193 patients (mean age: 59.7 ± 11.9, male: 135) were enrolled in this study and investigated. In all patients, the surgeon performed the UOLD uniformly on the right side between C3 and C6 vertebrae. To compare the surgical outcomes, patients were divided according to their dominant compressive side on the CT/MRI axial images at the most severe stenotic level. Also, patients were divided into the dominant myelopathy/radiculopathy symptom side according to the preoperative medical records.

Patients were divided into 3 groups according to the dominant compressive side: Group A (right side), Group B (symmetric), Group C (left side). The dominant compressive side was categorized, based on the side with definite spinal cord compression by using preoperative cervical spine axial CT/MRI image and significant compression. Also, patients were divided into 3 groups according to their dominant myelopathy symptom side: Group I (right side), Group II (both or symmetric), Group III (left side). The dominant myelopathy symptom side was determined by whether myelopathy symptoms or signs, such as hand clumsiness, gait disturbance, grip & release, finger escape sign and/or pathologic reflex were shown symmetrically or asymmetrically. Lastly, the patients were divided into 3 groups according to their dominant radiculopathy symptom side: Group X (right side), Group Y (symmetric), Group Z (left side). The dominant radiculopathy symptom side was categorized according to the patients’ complains of symptom. All categorization was performed by two orthopedic surgeons according to the patients’ medical records. If categorizations differed, a senior surgeon made the final decision.

### Radiographic measurements

Cervical spine lateral radiography with flexion and extension views was taken before and after surgery and at the last follow-up. Sagittal parameters including C2–7 range of motion (ROM) on flexion–extension lateral X-rays, C0–2 and C2–7 Cobb’s angle on the standing lateral radiograph and C2–7 sagittal vertical axis (SVA, distance from center of C2 vertebral body to posterior-superior corner of C7 vertebral body) were measured and compared among the groups. All digital radiographs were magnified and evaluated using a picture archiving communication system (PACS, INFINITT) and were measured before surgery, at 1 month after surgery and at the last follow-up.

### Clinical outcomes assessment

To evaluate the clinical outcomes, Neck Visual Analog Scale (VAS), Arm VAS, Japanese Orthopaedic Association (JOA) score and Neck Disability Index (NDI) score were obtained and evaluated. The JOA recovery rate also calculated and compared according to the previous formula. All clinical measurements were assessed before surgery, 1 month after surgery and at the last follow-up.

### C5 nerve root palsy assessment

Postoperative C5 root palsy was defined as a decline of one or more grade in deltoid and/or biceps muscle power occurred after surgery. Muscle power was determined by manual muscle testing. The confirmation of the patients with postoperative C5 root palsy was done by the senior author.

### Surgical techniques

**Figure 1:**
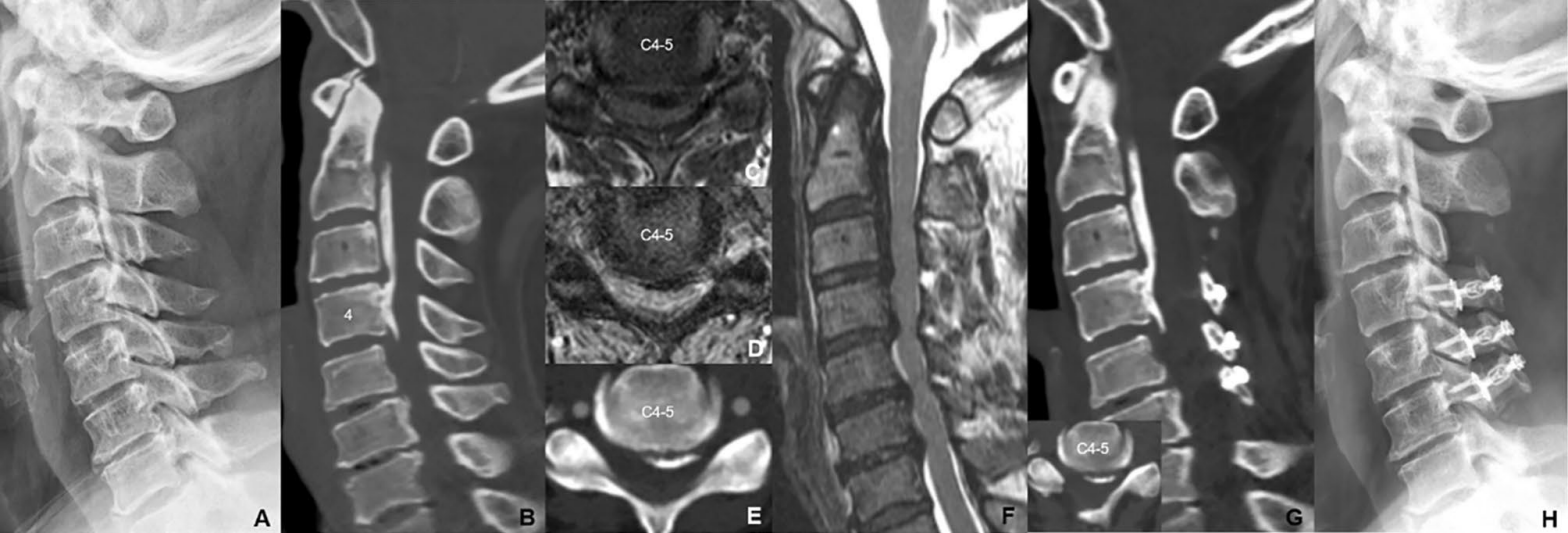
A 57-years female patient with myeloradiculopathy symptoms. Her chief complaints were posterior neck pain, right-side dominant arm pain, left-side dominant hand weakness, writing/chopsticks difficulty and gait disturbance. She had mixed type OPLL on cervical spine (**A**, **B**). Most significant cord compression was shown C4–5 and the OPLL mass was located to the central to left (**C**, **D**, **E**, **F**). Rt-sided open door laminoplasty C4–6 and total laminectomy C3 with C2 dome-like laminoplasty were performed (**G**) and the cervical alignment was maintained until postoperative 4 years (**H**). Preoperative clinical scores (JOA: 13 and NDI 23) were significantly improved at the last follow-up (JOA: 17 and NDI 7).

The standard posterior midline approach was performed on the proper level of each patient. The paravertebral muscles were detached, and the spinous processes were removed. To expand the central spinal canal and relieve cord compression, the UODL was performed according to the modified Hirabayashi method^1^ between C3 and C6 vertebrae. The lamina opening of the UOLD was done uniformly at the patients’ right-side in all cases. Right side gutter at the junction of the lamina and facet joint was cut completely by a high-speed burr and used as an opening side for all patients. Left side gutter was partially cut preserving the far cortex and formed a hinge side. After the right side-opening procedure, 10 mm-allobone (Laminoplasty Spacer, L&C BIO Inc., Seoul, Republic of Korea) and titanium mini-plate were fixed using mini-screws at the opened space. If there was neural foraminal stenosis, posterior foraminotomy was performed before the lamina opening procedures using the previously reported techniques^10^. The neural foraminal stenosis was defined as a foramen narrower than 50% of the normal contralateral side on CT axial images. In case of bilateral foraminal stenosis, neural foraminal stenosis was defined as a foramen narrower than 50% of the mean of the above and below cervical foramina^11^. All surgery was performed by a single surgeon (K.C.K.) at our institution (Fig. 1).

### Statistical analysis

Statistical analysis was performed using SPSS software (version 25.0. IBM Corp, Armonk, NY). To compare the radiographic measurements and clinical outcomes among the three groups, repeated-measures analysis of variance test and the Kruskal–Wallis test were used. Post hoc analysis was performed with the Tukey test and Mann–Whitney test. Chi-square test and Fisher exact test were used to compare the occurrence of C5 palsy among the three groups. The statistical significance was considered as *p* value < 0.05.

## Results

### Patients’ demographics

The average patient age was 59.7 ± 11.9 years and the mean follow-up period was 38.1 ± 15.1 months. The patients were sub-grouped on the perspective of dominant compressive side (Group A: 40, Group B: 120 and Group C: 33), dominant myelopathy symptom side (Group I: 29, Group II: 136 and Group III: 28) and dominant radiculopathy symptom side (Group X: 77, Group Y: 35 and Group Z: 81).

### Comparisons among 3 groups according to dominant compressive side

Preoperative radiographic and clinical parameters among 3 groups were comparable. There were no significant differences in radiographic (C0–2 angle, C2–7 angle and C–SVA) and clinical (neck VAS, arm VAS, JOA core and NDI score) parameters among 3 groups at postoperative 1 month and at the last follow-up period. The C5 palsy occurred in 3 (1.6%), 7 (3.6%), and 2 (1.0%) patients in each group, respectively, but were not significantly different (Tables 1, 2).

**Table 1:** Patient characteristics based on dominant compressive side.

	Group A (Right)	Group B (both/symmetric)	Group C (Left)
Number of patients	40	120	33
Female/Male	10/30	39/81	9/24
Age (years)	58.9 ± 11.7	60.5 ± 12.0	57.4 ± 10.9
Diagnosis			
CSM/HIVD	23	74	18
OPLL	17	46	15
Follow-up (months)	37.9 ± 16.9	38.1 ± 16.6	37.5 ± 15.7
BMI (kg/m^2^)	25.7 ± 4.4	24.6 ± 3.0	25.3 ± 3.3
Operative time (min.)	209.6 ± 45.3	203.7 ± 66.7	219.2 ± 68.1
EBL (mL)	353.8 ± 117.3	288.9 ± 203.8	298.5 ± 184.4

*CSM* indicated cervical spondylotic myelopathy; *HIVD* herniated intervertebral disc; *OPLL* ossification of posterior longitudinal ligament; *BMI* body mass index; *EBL* estimated blood loss.

**Table 2:** Comparison of outcomes according to dominant compressive side.

Parameters	Group I (Right)	Group II (Symmetric)	Group III (Left)	ANOVA *p* value	*p* value (I: II)	*p* value (II: III)	*p* value (III: I)
C2–7 ROM (°)							
Preoperative	43.7 ± 14.98	42.8 ± 13.37	42.2 ± 13.49	0.979	0.979	0.087	0.224
Postoperative	33.4 ± 10.41	32.4 ± 10.71	32.4 ± 11.17	0.757	0.476	0.968	0.757
Last f/u	33.9 ± 11.02	35.6 ± 11.7	29.1 ± 12.47	0.271	0.474	0.119	0.271
C0–2 angle (°)							
Preoperative	17.4 ± 6.86	16.0 ± 8.32	16.5 ± 9.43	0.457	0.221	0.892	0.457
Postoperative	18.7 ± 7.56	18.4 ± 8.78	19.4 ± 8.35	0.938	0.819	0.733	0.938
Last f/u	17.7 ± 6.32	18.5 ± 8.38	22.3 ± 8.87	0.321	0.868	0.158	0.321
C2–7 angle (°)							
Preoperative	12.4 ± 7.74	15.7 ± 9.38	14.2 ± 8.42	0.192	0.074	0.494	0.192
Postoperative	9.5 ± 7.07	13.9 ± 8.82	10.1 ± 6.72	0.011	0.008	0.051	0.011
Last f/u	9.7 ± 5.51	12.7 ± 8.5	12.8 ± 7.27	0.457	0.292	0.847	0.457
C-SVA (mm)							
Preoperative	23.3 ± 13.78	23.8 ± 13.75	23.1 ± 12.66	0.982	0.865	0.909	0.982
Postoperative	26.9 ± 12.44	26.6 ± 15.78	22.3 ± 8.82	0.434	0.492	0.350	0.434
Last f/u	22.3 ± 9.98	24.5 ± 12.74	27.7 ± 12.81	0.556	0.856	0.345	0.556
VAS (neck) score							
Preoperative	6.0 ± 0.63	6.1 ± 1.03	6.0 ± 0.89	0.065	0.024	1.000	0.065
Postoperative	2.1 ± 2.41	1.8 ± 1.87	2.2 ± 1.89	0.680	0.997	0.345	0.680
Last f/u	2.3 ± 2.46	1.5 ± 2.19	2.0 ± 1.75	0.237	0.187	0.185	0.237
VAS (arm) score							
Preoperative	6.6 ± 1.07	6.3 ± 1.4	6.3 ± 0.99	0.786	0.626	0.755	0.786
Postoperative	1.7 ± 1.8	2.0 ± 2.12	1.1 ± 1.26	0.264	0.691	0.106	0.264
Last f/u	1.9 ± 2.31	1.4 ± 2.05	2.5 ± 2.21	0.115	0.443	0.037	0.115
JOA score							
Preoperative	8.0 ± 0.76	8.7 ± 1.06	8.3 ± 0.98	0.133	0.060	0.284	0.133
Postoperative	12.9 ± 2.67	13.3 ± 3.1	13.6 ± 2.87	0.566	0.447	0.621	0.566
Last f/u	12.9 ± 2.84	14.1 ± 2.86	12.7 ± 3.54	0.279	0.198	0.246	0.279
JOA recovery rate	70.8 ± 22.8	76.2 ± 21.0	70.8 ± 22.8	0.437	0.246	0.502	0.719
NDI score							
Preoperative	21.9 ± 5.49	21.2 ± 4.27	20.2 ± 4.11	0.907	0.704	0.936	0.907
Postoperative	14.9 ± 7.49	12.6 ± 6.33	13.3 ± 5.25	0.238	0.102	0.506	0.238
Last f/u	12.8 ± 5.83	9.5 ± 5.96	12.8 ± 7.08	0.135	0.086	0.190	0.135
C5 palsy (Total)	3 (1.6%)	7 (3.6%)	2 (1.0%)	0.345^a^			

*C-SVA* indicated cervical sagittal vertical axis; *VAS* visual analog scale; *JOA* Japanese orthopaedic association; *NDI* neck disability index.

*Statistically significant (*p* value < 0.05).

^a^Chi-square test.

### Comparisons among 3 groups according to dominant myelopathy symptom side

There were no significant differences of radiographic and clinical parameters among 3 groups before surgery and at the last follow-up. However, at postoperative 1 month, mean neck VAS was slightly lower in Group I with right myelopathy symptom (0.8 ± 1.1) than in Group II with symmetric myelopathy symptom (2.0 ± 2.1) (*p* = 0.015) and in Group III with left myelopathy symptom (2.3 ± 1.7) (*p* = 0.019). The C5 palsy occurred 2 (1.0%), 7 (3.6%) and 3 (1.6%) patients in each group, respectively, but were not significantly different (Tables 3, 4).

**Table 3:** Patient characteristics based on dominant myelopathy symptom side.

	Group I (Right)	Group II (both/symmetric)	Group III (Left)
Number of patients	29	136	28
Female / Male	9/20	40/96	10/18
Age (years)	59.2 ± 11.0	60.4 ± 12.4	57.3 ± 9.3
Diagnosis			
CSM/HIVD	19	79	17
OPLL	10	57	11
Follow-up (months)	39.5 ± 13.3	36.5 ± 126.7	38.6 ± 17.8
BMI (kg/m^2^)	24.7 ± 3.3	24.9 ± 3.4	25.5 ± 3.4
Operative time (min)	205.9 ± 49.4	206.6 ± 67.3	213.8 ± 53.9
EBL (mL)	346.6 ± 184.1	303.5 ± 196.8	269.6 ± 202.4

*CSM* indicated cervical spondylotic myelopathy; *HIVD* herniated intervertebral disc; *OPLL* ossification of posterior longitudinal ligament; *BMI* body mass index; *EBL* estimated blood loss.

**Table 4:** Comparison of outcomes according to dominant myelopathy symptom side.

Parameters	Group I (Right)	Group II (Symmetric)	Group III (Left)	ANOVA *p* value	*p* value (I: II)	*p* value (II: III)	*p* value (III: I)
C2–7 ROM (°)							
Preoperative	43.7 ± 14.98	42.8 ± 13.37	42.2 ± 13.49	0.394	0.204	0.506	0.394
Postoperative	31.3 ± 9.7	32.8 ± 11.35	32.3 ± 8.61	0.852	0.649	0.855	0.540
Last f/u	31.6 ± 10.25	33.5 ± 12.12	35.2 ± 11.84	0.886	0.808	0.737	0.560
C0–2 angle (°)							
Preoperative	14.1 ± 8.11	16.5 ± 8.33	18.6 ± 8.09	0.161	0.226	0.233	0.045
Postoperative	16.2 ± 9.07	18.7 ± 8.36	20.9 ± 8.25	0.180	0.169	0.308	0.081
Last f/u	16.6 ± 8.32	17.7 ± 8.82	20.8 ± 8.5	0.744	0.887	0.485	0.462
C2–7 angle (°)							
Preoperative	13.1 ± 9.25	15.6 ± 9.13	12 ± 6.69	0.097	0.130	0.075	0.928
Postoperative	9.4 ± 6.86	12.2 ± 8.94	10.6 ± 6.25	0.089	0.061	0.242	0.466
Last f/u	10.2 ± 7.6	12.1 ± 6.2	9.4 ± 6.23	0.787	0.499	0.991	0.581
C-SVA (mm)							
Preoperative	23.5 ± 13.86	24.5 ± 14.14	20.9 ± 10.78	0.614	0.799	0.326	0.560
Postoperative	26.5 ± 14.71	26.8 ± 15.28	24.7 ± 10.98	0.915	0.983	0.682	0.735
Last f/u	28.5 ± 14.77	27.2 ± 14.42	26.7 ± 13.36	0.773	0.355	0.840	0.291
VAS (neck) score							
Preoperative	6.2 ± 0.76	6.2 ± 1.12	5.8 ± 1.05	0.482	0.909	0.252	0.482
Postoperative	1.8 ± 2.04	1.9 ± 1.98	2.4 ± 1.97	0.392	0.818	0.198	0.250
Last f/u	2 ± 1.8	1.6 ± 2.17	2.2 ± 2.55	0.419	0.250	0.390	0.919
VAS (arm) score							
Preoperative	6.4 ± 1.07	6.4 ± 1.29	5.9 ± 1.38	0.416	0.802	0.193	0.397
Postoperative	0.8 ± 1.1	2.0 ± 2.1	2.3 ± 1.7	0.015*	0.019*	0.238	0.004*
Last f/u	1.8 ± 2.38	1.6 ± 2.08	2.1 ± 2.18	0.703	0.877	0.392	0.631
JOA score							
Preoperative	8.1 ± 0.88	8.6 ± 1.07	9.0 ± 0.82	0.191	0.219	0.286	0.191
Postoperative	12.5 ± 2.94	13.3 ± 3.07	13.8 ± 2.3	0.365	0.218	0.660	0.185
Last f/u	12.7 ± 3.28	13.7 ± 3.04	14.3 ± 2.05	0.549	0.333	0.879	0.232
JOA recovery rate	72.4 ± 19.5	76.1 ± 22.0	72.4 ± 19.5	0.602	0.492	0.492	0.996
NDI score							
Preoperative	22.6 ± 3.37	20.9 ± 4.77	19.9 ± 2.48	0.353	0.310	0.557	0.072
Postoperative	13.8 ± 6.87	13.3 ± 6.48	13.1 ± 6.52	0.977	0.878	0.908	0.830
Last f/u	9.8 ± 6.96	11 ± 6.3	9.5 ± 5.37	0.628	0.417	0.533	0.731
C5 palsy (Total)	2 (1.0%)	7 (3.6%)	3 (1.6%)	0.779^a^			

Significant are in value [bold].

*C-SVA* indicated cervical sagittal vertical axis; *VAS* visual analog scale; *JOA* Japanese orthopaedic association; *NDI* neck disability index.

^a^Chi-square test.

*Statistically significant (*p* value < 0.05).

### Comparisons among 3 groups according to dominant radiculopathy symptom side

There were no significant differences of radiographic and clinical parameters among 3 groups before surgery and at the last follow-up. However, at postoperative 1 month, mean neck VAS was slightly lower in Group X (right radiculopathy symptom, 1.5 ± 1.5) than in Group Z (left radiculopathy symptom, 2.4 ± 1.2) (*p* = 0.043). The C5 palsy occurred 5 (2.6%), 6 (3.1%), and 1 (0.5%) patients in each group, respectively, but were not significantly different (Tables 5, 6).

**Table 5:** Patient characteristics based on dominant radiculopathy symptom side.

	Group X (Right)	Group Y (symmetric)	Group Z (Left)
Number of patients	77	35	81
Gender			
Female	28	6	25
Male	49	29	56
Age (years)	60.9 ± 11.03	58.11 ± 14.90	59.4 ± 11.23
Diagnosis			
CSM/HIVD	40	19	52
OPLL	37	16	29
Follow-up (months)	37.6 ± 16.60	36.2 ± 17.61	40.6 ± 15.71
BMI (kg/m^2^)	25.2 ± 3.45	24.4 ± 3.90	24.9 ± 3.20
Operative time (min)	207.6 ± 58.69	211.3 ± 67.17	205.9 ± 65.62
EBL (mL)	337.7 ± 191.28	277.9 ± 226.45	285.8 ± 184.09

*CSM* indicated cervical spondylotic myelopathy; *HIVD* herniated intervertebral disc; *OPLL* ossification of posterior longitudinal ligament; *BMI* body mass index; *EBL* estimated blood loss.

**Table 6:** Comparison of outcomes according to dominant radiculopathy symptom side.

Parameters	Group X (Right)	Group Y (both/symmetric)	Group Z (Left)	ANOVA *p* value	*p* value (I: II)	*p* value (II: III)	*p* value (III: I)
C2–7 ROM (°)							
Preoperative	40.8 ± 13.95	42.5 ± 14.89	41.3 ± 14.42	0.488	0.830	0.999	0.766
Postoperative	31.9 ± 9.66	35.3 ± 13.71	30.3 ± 11.48	0.628	0.320	0.235	0.983
Last f/u	31.1 ± 11.0	36.1 ± 14.64	31.9 ± 12.33	0.239	0.112	0.259	0.823
C0–2 angle (°)							
Preoperative	16.4 ± 8.57	14.6 ± 7.75	17.2 ± 8.27	0.319	0.550	0.286	0.822
Postoperative	19.9 ± 9.54	18.8 ± 9.1	20.2 ± 8.27	0.753	0.831	0.735	0.977
Last f/u	18.3 ± 8.9	15.6 ± 8.22	18.7 ± 8.75	0.352	0.279	0.183	0.955
C2–7 angle (°)							
Preoperative	13.9 ± 8.99	17.9 ± 10.23	14.2 ± 8.50	0.052	0.145	0.111	0.860
Postoperative	10.2 ± 7.6	12.6 ± 8.89	11.7 ± 7.44	0.243	0.275	0.844	0.423
Last f/u	13.0 ± 11.01	15.1 ± 8.35	12.2 ± 8.06	0.184	0.521	0.282	0.852
C-SVA (mm)							
Preoperative	25.4 ± 13.79	21.7 ± 15.69	23.3 ± 12.63	0.368	0.385	0.843	0.580
Postoperative	30.6 ± 15.55	29.4 ± 19.17	29.3 ± 14.66	0.853	0.919	0.999	0.857
Last f/u	27.8 ± 14.44	26 ± 14.06	27.5 ± 14.33	0.786	0.813	0.867	0.989
VAS (neck) score							
Preoperative	6.2 ± 1.1	5.9 ± 1.24	6.3 ± 0.89	0.661	0.834	0.637	0.958
Postoperative	1.5 ± 1.5	2.4 ± 2.20	2.5 ± 2.20	0.034*	0.140	0.990	0.043*
Last f/u	2.0 ± 2.3	1.1 ± 1.60	1.7 ± 2.13	0.430	0.396	0.633	0.849
VAS (arm) score							
Preoperative	6.0 ± 1.20	6.6 ± 1.65	6.4 ± 1.09	0.387	0.408	0.932	0.527
Postoperative	2.1 ± 2.04	2.8 ± 2.07	2.3 ± 2.17	0.523	0.492	0.652	0.915
Last f/u	1.5 ± 1.65	1.7 ± 2.62	1.9 ± 2.38	0.728	0.945	0.971	0.706
JOA score							
Preoperative	8.6 ± 1.00	8.6 ± 1.20	8.8 ± 1.04	0.686	0.999	0.803	0.710
Postoperative	13.2 ± 3.33	14.0 ± 2.73	13.0 ± 2.90	0.383	0.556	0.349	0.921
Last f/u	14.3 ± 2.77	14.5 ± 2.28	14.9 ± 1.79	0.308	0.946	0.626	0.297
JOA recovery rate	74.7 ± 24.1	73.8 ± 22.4	75.7 ± 19.13	0.906	0.983	0.906	0.957
NDI score							
Preoperative	22.6 ± 4.79	20.2 ± 4.23	20.2 ± 3.80	0.136	0.256	0.999	0.162
Postoperative	15.8 ± 5.45	14.4 ± 5.78	15.4 ± 7.40	0.710	0.688	0.802	0.949
Last f/u	10.5 ± 6.50	12.6 ± 6.39	10.3 ± 5.97	0.611	0.654	0.592	0.991
C5 palsy (Total)	5 (2.6%)	1 (0.5%)	6 (3.1%)	0.723^a^			

Significant are in value [bold].

*C-SVA* indicated cervical sagittal vertical axis; *VAS* visual analog scale; *JOA* Japanese orthopaedic association; *NDI* neck disability index.

*Statistically significant (*p* value < 0.05).

^a^Chi-square test.

## Discussion

When performing the UODL, surgeons usually open severe compressive side or dominant symptomatic side and if the symptom or cord compression is not one-sided, the lamina opening side is generally chosen based on surgeons’ convenience. But we found that the surgical outcomes did not seem to vary significantly depending on the lamina opening side. Then since March 2015, our institution uniformly performed the UODL only at the right side in all patients regardless of severe cord compressions or symptoms. 10 mm-lamina opening using the allobone spacer and mini-plate was performed for all patients consistently at the right side. Proper posterior foraminotomy was performed on both sides if there was a neural foraminal stenosis according to the CT axial images. We investigated whether the dominant compressive or symptomatic side affected postoperative surgical outcomes which include clinical (Neck-VAS, Arm VAS, JOA score, JOA recovery rate, NDI score, etc.) and radiographic (C2–7 ROM, C0–2 and C2–7 Cobb’s angle, C2–7 SVA and so on) parameters, as well as the occurrence rates of C5 palsy. Then we found that postoperative radiographic and clinical outcomes were not significantly different according to the dominant compressive or symptomatic side at the last follow-up.

Since the UODL was first introduced by Hirabayashi in 1983^2^, UODL is one of the most widely used techniques for patients with multi-level cervical myelopathy, along with the double door laminoplasty^4^ and surgical outcomes between the UODL and the double door laminoplasty are comparable in the literature^3,6,12^. Among these surgical procedures, unlike the double door laminoplasty, the UODL was performed by opening one side of the lamina (right or left). However, there was no guideline for selection of the lamina opening side. Although there are many studies for comparison of the surgical outcomes between the UODL versus the double door laminoplasty or the laminoplasty versus the laminectomy fusion, until now there are few reports for the selection of the opening side of laminoplasty in the UODL.

Until now, there were only two papers that suggested proper choice of the opening side in the UODL with detailed radiographic and clinical outcomes. Tang et al. and Shao et al.^8,9^ investigated preoperative radiographic parameters of the patients with the cervical OPLL and concluded that contralateral side opening is preferable to the ipsilateral side opening for lateral type of the cervical OPLL in the UODL. In both studies, they showed higher JOA recovery rates in the contralateral side opening group, especially with the sharp lateral OPLL mass than in the ipsilateral side opening, but there are some important information missing. First, there were no established criteria of the authors themselves for selecting the opening side in the UODL. It seems to be possible that they inadvertently selected the opening side to alleviate a more compressive or symptomatic area. Second, in general, the patients with cervical myelopathy occasionally show dominant radiculopathy or myelopathic symptoms on one side. In the clinical field, surgeons usually select more symptomatic sides in the UODL without clear evidence. Evaluation of preoperative symptoms is of significant clinical importance. However, in these two studies, they did not analyze the preoperative symptomatic side and did not analyze its effects on postoperative surgical outcomes. Finally, there was little data regarding postoperative C5 palsy according to the opening side in the UODL. From a clinical perspective, the evaluation of the effect of the opening side on the occurrence of the C5 palsy is very important. Because of these reasons, we think that the results from these two papers have some limitations in clinical evaluation.

Interestingly, at the postoperative 1 month, Group I (dominant preoperative myelopathy symptom in right side) showed slightly lower mean VAS (arm) score than other Groups and Group X (dominant preoperative radiculopathy symptom in right side) showed slightly lower mean VAS (neck) score than other Groups. Although postoperative radiographic and clinical outcomes were not significantly different among the groups at the last follow-up, these results are thought to mean that opening the same direction in the case of one-sided symptoms before surgery will partially help improve neck pain or arm pain immediately after surgery. The authors thought that the inflammatory materials that cause pain would have been washed out better on the lamina opening side. However, these immediately postoperative differences among the groups didn’t seem to be big and gradually decreased over time, resulting in no differences at the last follow-up.

According to previous literature, about half of the patients with cervical myelopathy had cervical radiculopathy simultaneously. Therefore, opening side of the UODL would have important effects for improvement or aggravation of radiating pain after the surgery. Lee et al.^10^ underwent lamina opening according to the direction of main symptoms and reported that the patients with additional posterior foraminotomy following the UODL showed significant improvement of VAS for arm pain compared with performing the UODL only without simultaneous foraminotomy. In this study, however, we uniformly performed the UODL on the right side as well as posterior foraminotomy in all patients if there was neural foraminal stenosis consistent with symptomatic radicular pain. The results were satisfactory regardless of the direction of preoperative radicular pain. These results show that the direction of the lamina opening side or dominant radicular symptom is not important, if proper posterior foraminotomy is accompanied in the UODL (Table 4).

Similarly with our study, although it was not a comparative study, Roselli et al. performed UODL only on the left side in 33 CSM patients and reported good clinical outcomes^13^. They developed a minimally invasive approach technique with unilateral muscle dissection and performed UOLD on the left side. This technique has advantages of the preserving of the contralateral muscular-ligament complex and reducing the risk of postoperative kyphosis and neck pain. But it has a limitation that proper foraminotomy on both side is impossible^14^. Unlike this study, we performed proper posterior foraminotomy on both side if needed and we conducted a comparative study.

C5 nerve root palsy after laminoplasty is known to be caused by excessive posterior shifting of the spinal cord. The incidence of C5 palsy after laminoplasty has been reported to vary from 2.3 to 16.7%^11,12^. Khuyagbaatar et al. conducted a biomechanical study and reported that C5 palsy after laminoplasty was more likely to occur when the OPLL is located on the lateral side rather than on the central side because of the imbalanced stress and tension on the C5 nerve root^15^. In our study, C5 palsy occurred in 6.2% (12/193) of patients, and all patients were recovered within 6 months after surgery. It occurred more frequently in case of lateral cord compression (group A and C, 6.8%) than in the case of symmetric cord compression (group B, 5.8%). However, the difference of each group was not statistically significant. Therefore, it was thought that lamina opening side according to the cord compression did not affect the occurrence of C5 palsy. We speculate that this is because appropriate foraminotomy was performed with the UODL. In the literatures, Kang et al. and Katsumi et al. reported that C5 palsy is induced by preexisting foraminal stenosis^16,17^ and Imagaya et al. reported that laminoplasty followed by foraminotomy could prevent C5 palsy by preventing tethering of the spinal cord through^18^.

There are some limitations in this study. First, it was a retrospectively designed study performed by a single surgeon. This is an inherent weakness of this study. Several uncontrolled variables and selection bias may exist. Second, we did not analyze the changes of cross-sectional area in cervical spinal canal, as shown in two recent reports. Nevertheless, this study is the first study to evaluate the effect of the lamina opening side for the surgical outcomes in the UODL, using the analysis of preoperative radiographic as well as clinical conditions with consistent right-sided lamina opening procedure. The authors are confident that the results of this study could help surgeons choosing the opening side of the lamina when performing the UODL.

## Conclusion

In UODL, the lamina opening side does not significantly affect postoperative radiographic and clinical outcomes including postoperative C5 palsy at more than 2 years follow-up. Therefore, when performing UODL, the choice of lamina opening side can be left to surgeon’s preference.

## Data Availability

All data analyzed during this study will be made available by the corresponding author on reasonable request.

## References

[1] Choi SH, Kang CN (2020). Degenerative cervical myelopathy: Pathophysiology and current treatment strategies. Asian Spine J..

[2] Hirabayashi K (1983). Expansive open-door laminoplasty for cervical spinal stenotic myelopathy. Spine Phila 1976.

[3] Heller JG, Raich AL, Dettori JR, Riew KD (2013). Comparative effectiveness of different types of cervical laminoplasty. Evid. Spine Care J..

[4] Kurokawa R, Kim P (2015). Cervical laminoplasty: The history and the future. Neurol. Med. Chir. Tokyo.

[5] Ma L (2018). Comparison of laminoplasty versus laminectomy and fusion in the treatment of multilevel cervical ossification of the posterior longitudinal ligament: A systematic review and meta-analysis. Med. Baltim..

[6] Okada M (2009). A prospective randomized study of clinical outcomes in patients with cervical compressive myelopathy treated with open-door or French-door laminoplasty. Spine Phila 1976.

[7] Chiba K (2006). Long-term results of expansive open-door laminoplasty for cervical myelopathy—Average 14-year follow-up study. Spine Phila 1976.

[8] Tang Y (2020). Choice of the open side in unilateral open-door laminoplasty for cervical ossification of the posterior longitudinal ligament. Spine Phila 1976.

[9] Shao T (2021). Modified axial computed tomography classification of cervical ossification of the posterior longitudinal ligament: Selecting the optimal operating procedure and enhancing the accuracy of prognosis. Quant. Imaging Med. Surg..

[10] Komagata M (2004). Prophylaxis of C5 palsy after cervical expansive laminoplasty by bilateral partial foraminotomy. Spine J..

[11] Lee SH (2016). Outcomes and related factors of C5 palsy following cervical laminectomy with instrumented fusion compared with laminoplasty. Spine Phila. 1976.

[12] Cho SK, Kim JS, Overley SC, Merrill RK (2018). Cervical Laminoplasty: Indications, Surgical Considerations, and Clinical Outcomes. J. Am. Acad. Orthop. Surg..

[13] Roselli R (2000). Open-door laminoplasty for cervical stenotic myelopathy: Surgical technique and neurophysiological monitoring. J. Neurosurg..

[14] Kothe R, Schmeiser G, Papavero L (2018). Open-door laminoplasty : What can the unilateral approach offer?. Oper. Orthop. Traumatol..

[15] Khuyagbaatar B, Kim K, Purevsuren T, Lee SH, Kim YH (2018). Biomechanical effects on cervical spinal cord and nerve root following laminoplasty for ossification of the posterior longitudinal ligament in the cervical spine: A comparison between open-door and double-door laminoplasty using finite element analysis. J. Biomech. Eng..

[16] Kang KC (2017). Preoperative risk factors of C5 nerve root palsy after laminectomy and fusion in patients with cervical myelopathy: Analysis of 70 consecutive patients. Clin. Spine Surg..

[17] Katsumi K, Yamazaki A, Watanabe K, Ohashi M, Shoji H (2013). Analysis of C5 palsy after cervical open-door laminoplasty: Relationship between C5 palsy and foraminal stenosis. J. Spinal Disord. Tech..

[18] Imagama S (2010). C5 palsy after cervical laminoplasty: A multicentre study. J. Bone Joint. Surg. Br..

